# Case Report: Polymyxin E-associated reversible leukopenia

**DOI:** 10.3389/fimmu.2025.1537972

**Published:** 2025-08-14

**Authors:** Suxin Wan, Jia-Quan Zhu, Lihua Wang

**Affiliations:** ^1^ Department of Pharmacy, Chongqing University Three Gorges Hospital, Chongqing University, Chongqing, China; ^2^ Fengdu General Hospital, Pharmaceutical Department, Chongqing, China; ^3^ Department of Endocrinology and Metabolism, Chongqing University Three Gorges Hospital, Chongqing University, Chongqing, China

**Keywords:** polymyxin E, polymyxin, leukopenia, Klebsiella pneumoniae, case report

## Abstract

**Introduction:**

With the increasing challenge of antibiotic resistance, polymyxin E is considered a last-line treatment option for infections caused by highly resistant bacteria. However, its use may lead to various adverse reactions, such as nephrotoxicity, neurotoxicity, and allergic reactions.

**Case Presentation:**

This study describes a case of polymyxin E-associated leukopenia in a 50-year-old female treated for *Klebsiella pneumoniae* infection. During polymyxin E therapy, the patient developed leukopenia, with white blood cell (WBC) counts declining from 5.65×10^9^/L to 0.91×10^9^/L. The condition resolved progressively after the cessation of polymyxin E. The Naranjo scale yielded a score of 7 for polymyxin E-associated leukopenia, while other medications scored ≤0. The WHO-Uppsala Monitoring Centre (WHO-UMC) causality classification system categorized the relationship as ‘probable.’

**Conclusion:**

These findings suggest that polymyxin E likely induces leukopenia, emphasizing the need for rigorous WBC monitoring during treatment and prompt discontinuation when hematologic abnormalities emerge to minimize patient health risks.

## 1 Introduction

Polymyxin E (also known as colistin), a bactericidal polypeptide antibiotic discovered in 1949, is used to treat Gram-negative bacterial infections (1). Its clinical use has resurged due to the increasing incidence of hospital-acquired infections caused by multidrug-resistant Gram-negative pathogens. Although initially employed in the 1950s, polymyxin E was largely phased out by the 1980s due to significant nephrotoxicity and neurotoxicity (2), resulting in limited pharmacological understanding of the agent. In recent years, it has been revived as a last-line therapeutic option for infections caused by multidrug-resistant Gram-negative bacteria—particularly Acinetobacter baumannii, Pseudomonas aeruginosa, and Enterobacteriaceae—driven by both escalating antimicrobial resistance and improved pharmacological insights (3). Nevertheless, the full spectrum of its adverse effects remains incompletely characterized.

Nation et al. have documented polymyxin E-induced nephrotoxicity (4, 5), with patients developing acute kidney injury within one week of intravenous administration. Notably, this condition is typically reversible upon discontinuation of treatment. The predominant clinical manifestations of polymyxin E-associated nephrotoxicity include renal tubular damage, decreased glomerular filtration rate, reduced creatinine clearance (Ccr), and elevated serum urea and creatinine concentrations. Dose-dependent nephrotoxicity remains the most frequently reported adverse event associated with intravenous polymyxin E therapy.

Intravenous polymyxin E may also induce neurotoxicity, though the precise mechanisms remain unclear. Current literature suggests the potential involvement of acetylcholine inhibition, impaired acetylcholine release, prolonged depolarization time, and calcium depletion (6). Recent case reports have highlighted cutaneous hyperpigmentation following polymyxin B therapy, potentially mediated through histamine-induced IL-6 overexpression in dermal tissues (7). Furthermore, a pharmacovigilance signal mining study using the U.S. FDA Adverse Event Reporting System (FAERS) database identified 918 polymyxin E-related adverse drug event (ADE) reports, encompassing 1,432 ADE occurrences. The most frequently reported ADEs were systemic disorders and administration site reactions (381 cases, 26.61%), followed by renal/urinary disorders (168 cases, 11.73%) and respiratory/thoracic/mediastinal disorders (125 cases, 8.73%). Hematologic/lymphatic system toxicities accounted for 94 cases (6.56%), including 15 leukopenia reports—representing only 3.42% of total ADEs (8). A similar case report documented a 5-year-old female patient who developed thrombocytopenia following colistin administration. Initially misdiagnosed as disease-related, the condition was ultimately attributed to drug-induced toxicity through pharmacovigilance assessment (9). Clinical evidence indicates that maintaining polymyxin dosages within appropriate ranges significantly reduces the incidence of related adverse reactions (10). The first PK/PD-based dosing recommendations for intravenous polymyxin E have now been globally adopted in hospital settings and have demonstrated effectiveness in optimizing therapeutic outcomes. In summary, the most frequently reported adverse reaction to polymyxin E remains nephrotoxicity. The rarity of hematotoxicity reports may have been overshadowed by the emphasis on nephrotoxicity, highlighting the need to increase public and clinical awareness of its potential hematologic effects.

Leukopenia is widely recognized to precipitate immune dysfunction, sepsis, septicemia, and aplastic anemia—significantly compromising patient quality of life. Reports of hematologic toxicity due to polymyxin E are rare, and greater awareness could help clinicians make more informed decisions. Furthermore, given the absence of established clinical guidelines for polymyxin E use, each clinical investigation and case report documenting its effects carries significant scientific value for practice optimization.

## 2 Case description

A 50-year-old female was admitted on August 18, 2024, with a 4-year history of elevated serum creatinine and recurrent fever over the preceding 5 days. Her medical history included type 2 diabetes mellitus, hypertension, chronic renal failure at the uremic stage, hyperphosphatemia, and hypothyroidism, all under standard therapeutic management. The patient had previously received metformin extended-release 0.5 g tid po for type 2 diabetes for three years, which was later switched to insulin glargine 28U qd due to suboptimal glycemic control. For hypertension with chronic kidney disease, captopril 25 mg tid po was initially prescribed but later replaced with valsartan 160 mg qd po following a severe dry cough. Two years prior, nocturnal hypertension prompted a change to amlodipine besylate 10 mg qd po. Subclinical hypothyroidism had been managed with levothyroxine 50 μg qd po for 12 months before being discontinued per physician evaluation. Her current medications (glucose-lowering/antihypertensive agents) were taken consistently, with no recent missed doses, although she reported suboptimal glycemic and blood pressure control. On physical examination, her height was 140 cm, weight 40 kg, body temperature (T) 39.5°C, pulse (P) 89 beats/min, respiratory rate (R) 20 breaths/min, and blood pressure (BP) 144/69 mmHg. The patient was alert and oriented, presenting with renal facies. No edema was observed in the facial, periorbital, or lower extremity regions. Cardiopulmonary auscultation was unremarkable. A functional left brachiocephalic arteriovenous fistula exhibited palpable thrills. Additional findings included mild diarrhea, fatigue, and cephalalgia. She reported no food/drug allergies and no family history of hematologic disorders. The diagnosis of admission was 1. Uremic stage of chronic renal failure: renal anemia, renal bone disease, renal hypertension; 2. Fever to be investigated: lung infection? Urinary tract infection? 3. Type 2 diabetes, type 2 diabetic nephropathy stage V; 4. Hyperphosphatemia; 5. Hypothyroidism.

On the day of admission, the patient stated: “The high fever has left me with extreme weakness, making it difficult to even sit up. The diarrhea is so frequent that I cannot leave the bed.” She exhibited significant anxiety regarding the persistent fever and diarrhea, expressing concern that the infection might be uncontrollable. Initial laboratory investigations revealed the following parameters: white blood cell count (WBC) 6.91×10^9^/L, neutrophil count (NE) 6.14×10^9^/L, hemoglobin 102 g/L, platelet count (PLT) 115×10^9^/L, C-reactive protein (CRP) 135.6 mg/L, procalcitonin (PCT) 22.87 ng/mL, serum creatinine (SCr) 538 μmol/L, random fingerstick blood glucose level of 9.6 mmol/L, and glycated hemoglobin (HbA1c) 6.3%. Non-contrast chest and abdominal CT imaging revealed left renal enlargement with perirenal fat stranding and focal septal thickening; a left-sided trace pleural effusion with pleural thickening; scattered solid and ground-glass micronodules in both lungs; minor inflammatory foci in the left lower lobe; a small pericardial effusion; pulmonary artery dilation; multiple mediastinal and bilateral axillary lymph nodes; right renal microlithiasis; and splenomegaly. The patient presented with recurrent high-grade fever. Based on laboratory and imaging findings suggestive of bloodstream infection, meropenem 1 g ivgtt q12h was initiated for anti-infective therapy. Intermittent hemodialysis was also implemented for uremia management.

On August 20, the patient continued to experience chills, fever (T 39.0°C), and diarrhea with yellow watery stools (over 10 episodes/day, without hematochezia, mucus, or purulent discharge), accompanied by reduced food intake and postprandial nausea and vomiting. The patient stated: “The diarrhea has made it impossible for me to eat anything. Every time I vomit, my throat burns intensely, and my whole body feels drained of energy.” At this stage, progressive physical decline and shaken confidence in treatment efficacy led to repeated inquiries to medical staff: “Will this infection ever improve?” Laboratory tests showed the following: CRP 166.3 mg/L, PCT 40.21 ng/mL, WBC 5.32×10^9^/L, NE 3.16×10^9^/L, and PLT 81×10^9^/L. Nucleic acid tests for 13 respiratory viruses and 5 tumor markers returned negative results. Blood cultures isolated Klebsiella pneumoniae. Antimicrobial susceptibility testing indicated extended-spectrum β-lactamases (ESBLs) negativity, with resistance limited to ampicillin and nitrofurantoin. Due to progressively elevated infection markers, septicemia and intestinal infection were suspected. Vancomycin 1 g ivgtt q12h was added to intensify anti-infective therapy. Concurrent interventions included intestinal flora modulation, acid suppression therapy, and antipyretic management.

On August 22, the patient remained febrile (T 37.6°C) with loose pasty stools (6 episodes/day, no hematochezia, mucus, or purulent discharge). The patient reported decreased appetite. Laboratory results were as follows: PCT 22.78 ng/mL, CRP 75.5 mg/L, WBC 5.63×10^9^/L, hemoglobin 78 g/L, and PLT 81×10^9^/L. Abdominal CT findings: 1. Multiple right renal calculi; left renal/perirenal inflammatory changes? 2. Heterogeneous gastric wall thickening with uneven density; edematous thickening of colorectal and partial small intestinal walls with reduced density—suggestive of inflammatory lesions? 3. Small-volume ascites and pelvic fluid; thickened and blurred peritoneum, omentum, and mesentery; 4. Enlarged lymph nodes in the bilateral inguinal regions, abdominal cavities, and retroperitoneum. Given the diagnosis of sepsis secondary to intestinal infection, and the observed decline in peak body temperature and infection-related biomarkers, the current anti-infective regimen was maintained.

On August 29, the patient continued to experience fever and diarrhea exacerbated by oral intake. Laboratory results showed PCT 4.94 ng/mL, CRP 26.9 mg/L, WBC 5.65×10^9^/L, NE 3.41×10^9^/L, hemoglobin 78 g/L, and PLT 105×10^9^/L. Stool microscopy revealed fungal elements and leukocytes 0-6/HP. Thoracoabdominal CT findings included: 1. Multiple right renal calculi, left perirenal fat stranding with focal septal thickening, and patchy hypodense lesions in the left kidney; 2. Bilateral pleural effusions increased compared to prior imaging; 3. Minor consolidation in the left lower lung lobe; 4. Enlarged lymph nodes in bilateral inguinal regions, abdominal cavity, and retroperitoneum; 5. Ascites and pelvic fluid, thickened peritoneum/omentum/mesentery; and a left lumbar triangle hernia. Infectious disease consultation recommended adjusting the anti-infective regimen to cefoperazone-sulbactam 3 g ivgtt q12h + tigecycline 100 mg ivgtt q12h, considering the recurrent diarrhea and abdominal CT findings indicative of intestinal infection.

On September 2, the patient continued to exhibit fever (T 39°C), chills, and diarrhea (yellow watery stools, 20 episodes/day), along with reduced food intake. Clostridioides difficile glutamate dehydrogenase antigen and toxin tests were negative. Laboratory parameters included PCT 45.56 ng/mL and CRP 88.2 mg/L. The patient was placed nil per os and provided with parenteral nutrition support.

On September 3, the patient remained febrile (T 38.3°C) with chills, poor appetite, and persistent diarrhea (yellow watery stools, five episodes/day). The patient reported: *“*Even when lying down, I feel like the world is spinning, with numbness in my hands and feet—I can’t even grip a water cup*.”* Family members described how the patient suffered from insomnia due to anxiety about worsening test results, frequently asking medical staff to explain the clinical significance of her laboratory values. Laboratory findings showed PCT 53.57 ng/mL, CRP 92.8 mg/L, WBC 5.65×10^9^/L, NE 3.26×10^9^/L, hemoglobin 72 g/L, and PLT 38×10^9^/L. Despite reduced stool frequency, the patient was transitioned to a liquid diet.

Given recurrent elevation of body temperature and infection biomarkers, presumed Gram-negative bacterial infection with suspected sepsis prompted escalation of antibiotic therapy to meropenem 0.5 g ivgtt q8h + polymyxin E methanesulfonate 150 mg ivgtt qd (loading dose 300 mg qd). Adjunctive therapies included intestinal flora modulation, antipyretic management, and thrombocyte-enhancing supportive care.

On September 4, the patient became afebrile. Laboratory results were as follows: PCT 5.82 ng/mL, CRP 109.1 mg/L, WBC 1.57×10^9^/L, NE 0.73×10^9^/L, hemoglobin 63 g/L, and PLT 9×10^9^/L. Metagenomic sequencing detected *Klebsiella pneumoniae* in blood samples, and the current therapeutic regimen was continued.

On September 6, the patient experienced persistent diarrhea (1–2 episodes/day, yellow watery stools) and reported generalized weakness, inability to ambulate independently, and dizziness. Laboratory tests revealed WBC 0.91×10^9^/L, NE 0.53×10^9^/L, hemoglobin 57 g/L, and PLT 9×10^9^/L. To clarify the cause of leukopenia, virological, nutritional, and autoimmune-related examinations were conducted. Results showed the following: Epstein–Barr virus (EBV) DNA <500 copies/mL; cytomegalovirus (CMV) DNA <200 copies/mL; and nucleic acids for influenza A, influenza B, and respiratory syncytial virus (RSV) were all negative. Vitamin B12 was 180 pg/mL; folate 3.2 ng/mL; ferritin 35 ng/mL; and serum albumin 38 g/L. Antinuclear antibody (ANA) was negative (titer <1:100), antineutrophil cytoplasmic antibody (ANCA) was negative, and the direct antiglobulin test (Coombs test) was also negative. A non-contrast head CT scan showed no definite abnormal density in the brain parenchyma. Mild widening of some cerebral sulci, fissures, and cisterns, along with dilation of the ventricular system, was observed. Midline structures remained centered, and no abnormalities were observed in the skull bones. The development of tri-lineage cytopenia (WBC/NE/PLT reduction) necessitated leukocyte/thrombocyte-enhancing therapies. Given the sustained efficacy of the ongoing anti-infective regimen, no treatment adjustments were made.

On September 9, the patient experienced worsening oral pain that significantly impaired eating and speech. The patient described: *“*Thick white patches have formed in my mouth, and swallowing feels like being cut by knives—even drinking water has become torture*.”* During this period, she became emotionally distressed and developed resistance to treatment, telling family members, *“*I don’t want to continue with the injections anymore*.”*

Laboratory results showed WBC 0.37×10^9^/L, NE 0.03×10^9^/L, hemoglobin 72 g/L, and PLT 30×10^9^/L. Bone marrow morphology analysis revealed rare, atypical lymphocytes, phagocytic histiocytes, a hypoplastic granulocytic lineage, and eosinophils accounting for 11.0%. A hematology consultation confirmed pancytopenia and recommended discontinuation of myelosuppressive agents.

Consequently, meropenem and polymyxin E were discontinued, and the regimen was switched to ceftazidime-avibactam 0.94 g iv qod (loading dose 2.5g) + voriconazole 0.2g po bid. A throat swab was collected for microbiological analysis, while transfusion support, leukocyte/thrombocyte-enhancing therapies, and glucocorticoid administration were continued.

On September 13, the patient remained afebrile. Complaints of dizziness, bloating, and poor appetite had improved, although she continued to pass loose, yellow, thin, pasty stools (six times/day). She reported subjective improvement: *“*The fever has finally subsided. Though still weak, my dizziness has lessened, and I can now drink some congee*.”* Family members noted that her emotional state had gradually stabilized, and she had begun to proactively inquire about rehabilitation plans.

Laboratory parameters showed PCT 17.29 ng/mL, CRP 7.7 mg/L, WBC 12.35×10^9^/L, and NE 10.15×10^9^/L. Fungal culture of the throat swab yielded Candida glabrata, and the current anti-infective regimen was maintained.

On September 29, the patient remained afebrile, with resolved diarrhea and stable mental status, and reported no somatic discomfort. Laboratory parameters included PCT 1.03 ng/mL, CRP 5.1 mg/L, WBC 5.19×10^9^/L, NE 2.34×10^9^/L, hemoglobin 80 g/L, and PLT 61×10^9^/L.

A non-contrast chest and abdominal CT scan demonstrated significant resolution of the previously observed focal inflammatory lesion in the left lower lobe, with only minimal residual fibrous strands and mild reticular changes remaining—without evidence of consolidation or ground-glass opacity. The previously scattered solid and ground-glass micronodules in both lungs had decreased in number and size (maximum diameter approximately 3 mm), with well-defined margins and no signs of new nodules or coalescence.

The bilateral minimal pleural effusions had completely resolved, with improvement in pleural thickening and no new effusions. The perinephric fat stranding around the left kidney showed improvement, with more homogeneous local density. Mediastinal and bilateral axillary lymph nodes had decreased in size (short axis <1 cm), some showing features of reactive hyperplasia. The pericardial space was clear, and pulmonary artery diameter was normal.

Overall clinical improvement was noted, although thrombocytopenia persisted. The patient was discharged per family request against medical advice.

Discharge diagnosis: 1, sepsis; 2, chronic renal failure uremic stage: renal hypertension, renal anemia, hyperphosphatemia; 3, intestinal infection; 4, urinary tract infection; 5, hypothyroidism; 6, pancytopenia; 7, chronic heart failure; 8, type 2 diabetes mellitus with multiple complications; 9, hypoproteinemia; 10, malnutrition; 11, gastrointestinal dysfunction. On October 30, outpatient follow-up evaluation showed WBC 3.61×10^9^/L, NE 1.82×10^9^/L, PLT 83×10^9^/L, and SCr 307 μmol/L. The patient reported no significant symptoms.

## 3 Discussion

The patient reported suboptimal glycemic and blood pressure control upon admission. However, clinical inquiry revealed consistent adherence to insulin glargine 28U qd and amlodipine besylate 10 mg po qd without missed doses. Admission laboratory tests showed a random capillary blood glucose level of 9.6 mmol/L, HbA1c of 6.3%, and BP 144/69 mmHg. Fasting blood glucose measured at 8:00 AM the following day was 5.8 mmol/L, and the postprandial glucose level was 8.9 mmol/L one hour after lunch. According to type 2 diabetes management guidelines—and considering the patient’s age—glycemic control was deemed essentially within the target range. As the patient maintained self-administered glucose-lowering and antihypertensive medications, no additional pharmacologic interventions were initiated during hospitalization. A comprehensive review of the patient’s electronic medical records enabled integration of historical diagnoses into the final discharge summary.

Preclinical studies indicate that early-stage diabetes induces structural and molecular alterations in bone marrow, including enhanced tibial adipogenesis preceding stem cell depletion in murine models. This has been correlated with suppression of transforming growth factor-β (TGFB) signaling (11). Nevertheless, direct clinical evidence linking diabetes to myelosuppression remains scarce. Acute diabetic metabolic syndromes (e.g., diabetic ketoacidosis) typically present with overt hyperglycemia, and current clinical guidelines do not recognize bone marrow suppression as a chronic complication of diabetes. In this case, the absence of temporal correlation between well-controlled glucose parameters and leukopenia further diminishes the likelihood of diabetes-induced hematopoietic dysfunction.

*In vitro* studies by Elena Della et al. demonstrated that the uremic microenvironment impairs osteogenic differentiation of bone marrow mesenchymal stromal cells (BMSCs), though this finding lacks validation in *in vivo* models (12). An observational study reported impaired marrow reconversion on MRI in stage 3–5 chronic kidney disease (CKD) patients, though no statistical correlation was observed between anemia and marrow reconversion (13). Another study noted that hematopoietic progenitor cells (BFU-E) in pre-dialysis uremic patients exhibited baseline growth suppression, whereas BFU-E proliferation in continuous ambulatory peritoneal dialysis (CAPD) patients approached near-normal levels under baseline conditions (14). These findings suggest that while uremia in advanced CKD may induce bone marrow dysfunction, dialysis intervention could partially restore marrow function.

Furthermore, the literature review revealed no documented cases of uremia-induced leukopenia via bone marrow suppression, necessitating further investigation into their potential association.

The Chinese Multidisciplinary Expert Consensus on Rational Clinical Use of Polymyxin Antimicrobials (2021) states that colistimethate sodium is primarily renally excreted and may accumulate in cases of renal impairment, requiring dose adjustment. However, during regular hemodialysis, sodium polymyxin E mesylate exhibits low volume of distribution and low protein binding, allowing efficient clearance via diffusion/convection. Consequently, the administration of a loading dose alone poses minimal risk of drug accumulation (15).

Enrico Fiaccadori et al. corroborated that colistin sodium mesylate is readily eliminated through dialysis/hemofiltration, with negligible accumulation potential (16). During hospitalization, the patient received intermittent hemodialysis regularly, though post-admission body weight and SCr measurements were omitted. Creatinine clearance (Ccr) calculated via the Cockcroft–Gault formula using baseline data, yielded 6.983 mL/min, consistent with end-stage renal disease. The Prato Polymyxin Consensus states that in patients with renal insufficiency who are on intermittent hemodialysis, the following dosing regimen should be used: on non-dialysis days, a 130 mg mucin-activated base (CBA)/day dose of colistin sodium mesylate should be given; on dialysis days, a supplemental dose of either 40 mg of colistin sodium mesylate or 50 mg of CBA should be given; and, if possible, a supplement to the baseline (non-dialysis) daily dose should be administered with the next regular dose at the end of the day. Intermittent hemodialysis should be performed as late as possible within the dosing interval to minimize the amount of colistin sodium mesylate and mucin-forming substances lost to the extracorporeal system (2). Given the hospital’s sodium polymyxin E methanesulfonate formulation (150 mg/vial) and the patient’s dialysis frequency (every 2–3 days), strict adherence to post-dialysis supplemental dosing (40–50 mg) would incur substantial financial burden and drug wastage (half a dose per administration). Consequently, a maintenance dose of sodium polymyxin E methanesulfonate 150 mg/day was implemented. Throughout treatment, polymyxin E dosing remained strictly within label specifications and consensus guidelines, with minimal accumulation risk. Therefore, the direct correlation between dose adjustment of polymyxin E and leukopenia is low.

The initial assessment was for a complete blood count reduction caused by polymyxin E. However, the thrombocytopenia in this case did not have a clear temporal association with polymyxin E. Persistent infection also limited the ability to effectively rule out infection-associated platelet consumption. Additionally, general bacterial infections do not typically cause a significant decrease in white blood cell counts. Based on the overall clinical pattern, the adverse reaction was ultimately synthesized as leukopenia.

Naranjo scale scores for evaluating causality (17) showed a likelihood score of 1 for meropenem and 2 for cefoperazone-sulbactam sodium—both categorized as “possible” associations with thrombocytopenia. The drug inserts supported this potential relationship. A prescription for meropenem (1 g ivgtt q12h) was initially issued on August 18 and discontinued on August 29. On September 3, the anti-infective regimen was adjusted to meropenem (0.5 g ivgtt q12h, halved from the previous dose) in combination with polymyxin E methanesulfonate (150 mg ivgtt qd), which was discontinued on September 9. The dosage remained unchanged during both meropenem treatment periods.

The patient had previously received meropenem monotherapy before the administration of Polymyxin E, during which thrombocytopenia occurred, but no leukocyte abnormalities were observed. Pancytopenia subsequently developed after the combination therapy of meropenem and polymyxin E was introduced. Studies have reported that meropenem has a high clearance rate during regular hemodialysis, and the risk of drug accumulation when used alone is low (18). According to the package insert of meropenem for injection (Sumitomo Pharma), meropenem-related hematotoxicity is generally mild and reversible. While the incidence and severity may increase when combined with other antimicrobial agents such as vancomycin, the reported hematologic effects are primarily limited to thrombocytopenia, with no known reports of other hematologic abnormalities. However, according to the Common Terminology Criteria for Adverse Events (CTCAE) Version 5.0 issued by the National Cancer Institute (NCI) (19), the leukopenia in this case was classified as grade 3 (severe), which is inconsistent with the mild nature mentioned above. Therefore, there is no significant association between meropenem and leukopenia. The Naranjo scale score also showed that the actual association score between meropenem and leukopenia was −2 (≤0, suspicious), indicating a low likelihood of causation. These combined factors ruled out meropenem as a likely cause of leukopenia, consistent with the conclusions of Raza et al. (20). In contrast, a recent pharmacovigilance study mining the U.S. FDA Adverse Event Reporting System (FAERS) identified 94 cases of polymyxin E-associated blood/lymphatic system toxicities, with leukopenia ranking 17th among reported adverse drug events (15 cases) (8). The authors emphasized the need for heightened clinical vigilance regarding polymyxin E-related hematologic toxicity.

Turkish scholar Serhan Kupeliy also documented a case of polymyxin E-induced thrombocytopenia in a young female with chronic renal failure, in whom platelet counts progressively normalized after discontinuation of polymyxin E (9). An *in vitro* study by Ahmed M. et al. confirmed that polymyxin E can induce cell death in human macrophage-like THP-1 and neutrophil-like HL-60 cells (21).

In this case, the patient developed leukopenia and neutropenia (**Table 1**) one day after receiving a polymyxin E infusion. During treatment, symptomatic therapies aimed at elevating white blood cell and platelet counts showed limited efficacy. However, the condition markedly improved three days after discontinuation of polymyxin E and initiation of supportive care. Therefore, polymyxin E was strongly suspected to be the cause of leukopenia. The Naranjo Adverse Drug Reaction Probability Score for polymyxin E was 7, categorizing the association as “probable” (**Table 2**), whereas all other medications received scores below 0. The WHO-UMC causality assessment system (22) similarly classified polymyxin E as “probable,” while all other agents were considered “unlikely.” These data establish a significant temporal and probabilistic association between polymyxin E and cytopenia. Additionally, Lexicomp^®^ database analysis (23) revealed no clinically relevant drug–drug interactions among the patient’s concurrent medications.

**Table 1:** Pathway diagram for the treatment plan of anti-infective drugs.

Date (2024)	WBC (3.5-9.5*10^9/L)	NE (1.8-6.3*10^9/L)	PLT (120-350*10^9/L)	Antimicrobial therapy regimen
Aug—18	6.91	6.14	115	Meropenem 1g ivgtt q12h.
Aug—20	5.63	3.61	81	Meropenem (1g ivgtt q12h) + Vancomycin (1g ivgtt q12h).
Aug—29	5.65	3.41	105	Stop meropenem+vancomycin and adjust to ceftazidime sulbactam (3g ivgtt q12h) + tigecycline (100mg ivgtt q12h).
Sep—3	5.65	3.26	38	Stop cefoperazone sulbactam + tigecycline, adjust to meropenem (0.5g ivgtt q8h) + polymyxin E sulfate (150mg ivgtt qd, first dose 300mg).
Sep—4	1.57	0.73	9	Meropenem (0.5g ivgtt q8h) + Polymyxin E Methanesulfonate (150mg ivgtt qd).
Sep—6	0.91	0.53	9	Meropenem (0.5g ivgtt q8h) + Polymyxin E Methanesulfonate (150mg ivgtt qd).
Sep—9	0.37	0.03	30	Discontinue meropenem and polymyxin E sulfate, and switch to ceftazidime-avibactam (0.94g iv qod, first dose 2.5g) + voriconazole (0.2g po bid).
Sep—13	12.35	10.15	30	Ceftazidime-avibactam (0.94g iv qod) + voriconazole (0.2g po bid).
Sep—29	5.19	2.34	61	Cessation of cefotaxime, avibactam and voriconazole, the patient is discharged.

WBC, white blood cell count; NE, neutrophilic granulocyte; PLT, platelet; iv, intravenous injection; ivgtt, intravenously guttae; q8h/q12h, take medication once every 8 hours/12hours; qd, quaque die; qod, quaqueomnidie/everyotherday.

**Table 2:** ADR probability scale.

To assess the adverse drug reaction, please answer the following questionnaire and give the pertinent score				
Assessment Items	Yes	No	Do not know	Score
1. Are there previous conclusive reports on this reaction?	+1	0	0	+1
2. Did the adverse event appear after the suspected drug was administered?	+2	–1	0	+2
3. Did the adverse reaction improve when the drug was discontinued or was a specific antagonist administered?	+1	0	0	+1
4. Did the adverse reaction reappear when the drug was readministered?	+2	–1	0	0
5. Are there alternative causes (other than the drug) that could on their own have caused the reaction?	–1	+2	0	+2
6. Did the reaction reappear when a placebo was given?	–1	+1	0	0
7. Was the drug detected in blood (or other fluids) in concentrations known to be toxic?	+1	0	0	0
8. Was the reaction more severe when the dose was increased or less severe when the dose was decreased?	+1	0	0	0
9. Did the patient have a similar reaction to the same or similar drugs in any previous exposure?	+1	0	0	0
10. Was the adverse event confirmed by any objective evidence?	+1	0	0	+1

ADR, adverse drug reaction.

ADR was assigned to a probability category from the total score as follows: definite ≥ 9, probable 5–8, possible 1–4, and doubtful ≤ 0.

In summary, the timely identification of drug-induced adverse reactions is critical for improving patients’ quality of life, yet achieving this requires more case reports to raise awareness among clinicians. In this case, the delayed recognition of polymyxin E-associated toxicity led to successive hematologic crises, underscoring the need for close monitoring of white blood cell levels during treatment and prompt discontinuation if abnormalities arise.

Given the absence of international guidelines for polymyxin E use, it is foreseeable that as its application expands in the treatment of multidrug-resistant Gram-negative infections, enhancing clinicians’ awareness of polymyxin E-induced hematologic toxicity will be essential for informed, evidence-based decision-making.

We acknowledge several limitations of this study. First, as a single case report, the findings cannot be generalized, despite the detailed documentation of the patient’s clinical course. Second, due to the patient’s complex comorbidities (e.g., diabetes, uremia), we could not entirely rule out their potential influence on the observed outcomes, despite careful, evidence-based analysis. Third, the direct mechanism of leukopenia was not explored due to insufficient evidence. Fourth, the case lacked baseline bone marrow biopsy data. Fifth, although the likelihood of tigecycline-induced myelosuppression was considered low, the possibility of confounding interference cannot be entirely excluded. Lastly, unrecognized factors—such as genetic predisposition, environmental exposures, or undetected metabolic abnormalities—may have indirectly affected the interpretation of results.

## 4 Conclusion

Polymyxin E may induce reversible leukopenia. When leukopenia occurs during polymyxin E therapy, clinicians should maintain vigilance and promptly evaluate potential for drug-induced causation. We recommend enhanced dynamic monitoring of multiple hematopoietic cell lines during polymyxin E administration, as well as additional clinical studies to facilitate evidence-based guideline development. These measures will help optimize the clinical use of this “last-resort” antimicrobial agent while balancing therapeutic efficacy with patient safety.

## Data Availability

The original contributions presented in the study are included in the article/supplementary material. Further inquiries can be directed to the corresponding authors.
